# Effects of *Tilletia foetida* on Microbial Communities in the Rhizosphere Soil of Wheat Seeds Coated with Different Concentrations of Jianzhuang

**DOI:** 10.1007/s00248-021-01696-w

**Published:** 2021-02-01

**Authors:** Ghulam Muhae Ud Din, Zhenzhen Du, Han Zhang, Sifeng Zhao, Taiguo Liu, Wanquan Chen, Li Gao

**Affiliations:** 1 State Key Laboratory for Biology of Plant Disease and Insect Pests, Institute of Plant Protection, Chinese Academy of Agricultural Sciences, Beijing, 100193 China; 2 Key Laboratory at Universities of Xinjiang Uygur Autonomous Region for Oasis Agricultural Pest Management and Plant Protection Resource Utilization, Shihezi University, Xinjiang, 832003 China

**Keywords:** Wheat common bunt, *Tilletia foetida*, Fungi community, Bacterial community

## Abstract

*Tilletia foetida* (syn. *T. laevis*) leads to wheat common bunt, a worldwide disease that can lead to 80% yield loss and even total loss of production, together with degrading the quality of grains and flour by producing a rotten fish smell. To explore the potential microbial community that may contribute to the control of soil- and seed-borne pathogens, in this study, we analyzed the effects of the plant pathogenic fungus *T. foetida* on rhizosphere soil microorganisms in wheat seeds coated with different concentrations of a fungicide (Jianzhuang) used to control the disease. To analyze the bacterial and fungal abundance in *T. foetida*-infected and mock-infected plants, the microorganisms were sequenced using high-throughput HiSeq 2500 gene sequencing. The results showed that bacterial communities, including *Verrucomicrobia*, *Patescibacteria*, *Armatimonadetes*, *Nitrospirae*, *Fibrobacteres*, *Chlamydiae*, and *Hydrogenedentes*, and fungal communities, including *Basidiomycota* and *Ciliophora*, were more prevalent in the mock group than in the *T. foetida*-infected group, which may contribute to the control of wheat common bunt. Moreover, cluster and PCoA analysis revealed that replicates of the same samples were clustered together, and these results were also found in the distance index within-group analysis for bacterial and fungal communities in the *T. foetida*-infected and mock groups.

**Supplementary Information:**

The online version contains supplementary material available at 10.1007/s00248-021-01696-w.

## Introduction

*Tilletia foetida* (syn. *T. laevis*) is a pathogen that can lead to wheat common bunt [1, 2]. Plants infected with *T. foetida* usually produce a lower yield, with low quality compared to healthy plants. Reductions in the quality and quantity of infected plants occur due to replacement of mature grains with bunt sori [1]. Additionally, wheat flour millers usually refuse grains infected by *T. foetida*, as very low infection rates can result in obvious unwanted odors in flour [3, 4]. The disease incidence can reach 70 to 80% using *T. foetida*-susceptible seeds, with 41% yield loss in Romania [5] and losses reaching 25–30% and 10–20% in Iran and Turkey, respectively [6].

Rhizosphere soil is a dynamic and complex environment; its biological activity is typically regulated by microorganisms, which play crucial roles in sustaining the health of agricultural and natural soil systems [7]. It is well known that fungal and bacterial communities are responsible for multifaceted biological functions in soils, and maintaining the biodiversity of microbes is crucial to soil fitness [8]. However, many factors affect rhizosphere soil microorganisms, such as plant pathogens, which are a critical component of rhizosphere microbial communities and play an important role in plant growth and health [9]. Some fungi are known for biocontrol activity against pathogenic microorganisms [10], which positively support plant productivity by enhancing plant growth. However, some fungi negatively influence plant health, such as some plant pathogens in the soil; for example, *Fusarium graminearum* can cause stalk rot disease of maize [11], *Verticillium nonalfalfae* can cause verticillium wilt on tree of heaven [12], and *Macrophomina phaseolina* can cause dry root rot disease [13]. *T. foetida* is a soil- and seed-borne pathogen that leads to wheat common bunt, which may cause 80% loss in China and degrade the quality of wheat seeds and flour by producing rotten fish smells. Moreover, the teliospores of the pathogen can live in soil for up to 10 years and can germinate or even undergo excretion by animals or people.

Seed treatments with fungicides are broadly used to control some fungal pathogens, such as *Alternaria alternata* and *Fusarium* sp. [14, 15]. *T. foetida* has the ability to penetrate locally into host plants, and seed treatment with fungicides is approved as an option for its control [16]. Common bunt symptoms appear during the boot stage under conducive environmental conditions and are difficult to control at this stage through fungicide spraying [17]. Therefore, fungicide application for seed treatment has been used for disease control. Previous studies showed that Jianzhuang fungicide has good results against different pathogens, such as *Pseudomonas*, compared to triazole, a common wheat fungicide, against different fungal pathogens [18–20]. However, pesticides often affect microorganisms in the soil based on the dose, properties of the soil, and environmental factors [21–23]. Some studies have shown that glyphosate application increased *Proteobacteria* concentrations in the rhizospheres of corn (*Zea mays*) and soybean (*Glycine max*) [24]. Ryan concluded that pesticide usage might affect some groups of microorganisms in the soil but has little effect on the soil community [25]. Gupta and Kalia [23] reported that the usage of pesticides affects all of the soil microbes, with reductions in the average populations of all groups studied in soil samples from fields with rice-wheat cropping. Regarding the use of Jianzhuang in controlling wheat common bunt, there is little information on the effects of fungicides on soil microorganisms.

To optimize the concentration of Jianzhuang applied on wheat seeds, based on Illumina HiSeq 2500 sequencing, this study characterized the rhizosphere fungal and bacterial communities in the context of infection with the plant pathogen *T. foetida*. Our work will lay a foundation for potential biocontrol activity with rhizosphere bacterial and fungal communities and provide insights into the interactions of plant pathogens, rhizosphere bacteria and fungi, and plant growth.

## Materials and Methods

### Plant Material and Pesticide Application

The wheat (*Triticum aestivum* L.) spring cultivar Morocco (highly susceptible) was obtained from the Institute of Plant Protection (IPP), Chinese Academy of Agricultural Sciences (CAAS), Beijing, China. Wheat seeds were sterilized with 30% sodium hypochlorite for 5 min, rinsed 5 times in ddH_2_O, and germinated for 30 days in an incubator (AUCMA, Qing Dao, China) to vernalize after fungicide application. The fungicide programs are illustrated in Table 1. After vernalization, seedlings were grown in a 1:2 mixture of organic matter (peat moss, Beijing, China) and soil (Beijing, China) in pots. Seedlings were grown in a 14-h light/10-h dark cycle at 15 °C to the tillering stage and at 25 °C at the boot stage.

**Table 1:** Jianzhuang application against *T. foetida* infected and mock in wheat rhizosphere

Treatments	Treatments (%)	Infected	Mock
2.25 mL/100 kg	1.5% of RD	IA	NG
4.5 mL/100 kg	3% of RD	IB	NH
7.5 mL/100 kg	5% of RD	IC	NI
150 mL/100 kg	RD	ID	NJ
225 mL/100 kg	1.5 time of RD	IE	NK
No seed treatment	–	IF	NL

RD stand for recommend dose of Jianzhuang for wheat. Infected means inoculation of *T. foetida* in the root zone of the plants, while mock means no application of *T. foetida*

### Fungal Inoculation

*T. foetida* was grown on 2% agar media and incubated for 15 days at 15 °C under 24 h of light. The mycelium of *T. foetida* was harvested under laminar flow by adding 5 mL of ddH_2_O onto each *T. foetida* culture plate. Hyphae of *T. foetida* were injected 5 times into the root zone at 2-day intervals, while mock plants were treated with ddH_2_O. The suspension contained infectious hyphae at a concentration of 10^6^ cfu/mL with an OD_600_ of 0.15. Rhizosphere soil was collected during the booting stage of wheat from *T. foetida*-infected and mock plants.

### Molecular Detection of the Infection of *T. foetida* in Inoculated Wheat Leaves

DNA was extracted from wheat leaves 1 week after *T. foetida* inoculation using a Plant Genomic DNA Kit (TianGen, China). The primer sequences for *T. foetida* were (5′-TCACTTCAAGGTCGTTCCCG-3′)/L60R (5′-CGGGTCGAGGGGCGTAAACTTGA-3′). Polymerase chain reaction (PCR) and gel electrophoresis were performed to visualize the expected 660-bp band of *T. foetida* with high specificity, as described by Yao et al. [4].

### Soil Sampling

Soil samples were collected from both *T. foetida*-infected and mock pots at 20 cm depth. For each sample, there were 6 *T. foetida*-infected plants and 6 mock plants, with 6 rhizosphere soil samples collected from each infected plant and 6 from mock plants. After shaking the roots to remove loose soil, a sterile brush was used to collect the residual soil from the roots. Equal amounts of rhizosphere soil from the three wheat plants were mixed and stored at − 80 °C. Ten grams of rhizosphere soil was collected and mixed into 90 mL of distilled water in a 150-mL tube (Houdior, China). These tubes were shaken for 10 min in an incubator shaker (ZHWY-200D, China) and incubated for 5 min. Soil solution (1 mL) for every sample was taken and sequenced after culturing.

### DNA Extraction and PCR Amplification

Soil microbial DNA was extracted using a HiPure soil DNA kit (Magen, Guangzhou, China) following the manufacturer’s protocols. DNA was extracted from 0.5 g of soil suspension. The concentration and quality of every extracted DNA sample were evaluated based on the 260/280 nm and 260/230 nm absorbance ratios obtained using a NanoDrop 2000 (Thermo Scientific, USA). With the primers 341F (5′-CCTAGGGNGGCWGCAG-3′) and 806R (5′-GGACTACHVGGGTATCTAAT-3′) for V3–V4 of the 16S RNA gene and ITS3_KYO2 (5′-GATGAAGAACGYAGYRAA-3′) and ITS4 (5′-TCCTCCGCTTATTGATATGC-3′) for ITS2 region of ribosomal DNA, PCRs were performed in triplicate with a total volume of 50 μL, including 5 μL of 10× KOD buffer, 5 μL of 2.5 mM dNTPs, 1.5 μL of each primer (5 μM), 1 μL of KOD polymerase, and 100 ng of template DNA. The amplification program was 95 °C for 2 min, followed by 27 cycles at 98 °C for 10 s, 62 °C for 30 s, and a final extension of 68 °C for 10 min.

### Illumina HiSeq 2500 Sequencing

PCR products were recovered using a 1.5% agarose gel and purified using an AxyPrep DNA Gel Extraction Kit (Axygen Biosciences, Union City, CA, USA) according to the manufacturer’s guidelines and quantified using an ABI Step OnePlus Real-time PCR system (Life Technologies, Foster City, USA). Purified amplicons were pooled in equimolar amounts and paired-end sequenced (2 × 250) on an Illumina platform according to standard protocols. Raw readings were stored in the NCBI Sequence Read Archive (SRA) database. Sequencing was performed in triplicate using samples from three independent experiments.

### Data Analysis

After sequencing, all raw reads were analyzed using the QIIME standard pipeline [26] to trim the low-quality reads. Operational taxonomic units (OTUs) were clustered with a 97% similarity cutoff using UPARSE [27] (version 7.1 http://drive5.com/uparse/). The taxonomic classification was further checked using the Ribosomal Database Project (RDP) classifier (version 2.2) based on the Greengenes Database with 0.8 to 1 confidence [28, 29]. With the cluster file, alpha diversity statistics, including the Chao1 richness estimate, Shannon diversity index, and Simpson diversity index were calculated in MOTHUR for each sample [30, 31]. Beta diversity statistics based on UniFrac metrics were also carried out, including cluster analysis, weighted UniFrac distance metrics, and principal component analysis (PCoA) [31, 32]. Cluster analysis (CA) was used to group communities of twelve different samples. Weighted UniFrac distance metrics were used to estimate the community diversity of twelve samples. PCoA analysis to compare the discrepancies among individuals and communities was conducted based on (1) taxonomy results from the NCBI database; (2) OTUs described above; and (3) weighted UniFrac metrics.

## Results

### Phylotypes and Diversity of the Microbial Community

The soil samples were obtained from infected and mock plants with different concentrations of Jianzhuang, which were verified by specific primer markers of *T. foetida* (Figure S1). Using Illumina HiSeq 2500 sequencing, raw reads were produced for these samples. After filtering the raw reads using the QIME standard pipeline and removing all other chimeric tags, effective tags were used for further analysis. Average, total, minimum, and maximum ratios of all samples for raw data, clean data, raw tags, clean tags, and effective tags were calculated with the effective ratio. In total, 83,483–106,580 effective tags were obtained for these samples. Additionally, the percentage of valid data for each sample was more than 88%, indicating that at least 88% of tags of each sample met the requirements of sequencing length and quality (Table S1).

Furthermore, the number of OTUs was calculated for bacterial communities with three replications for each sample, and the changes in species abundance were also calculated from the domain to species level. OTU results showed that there were different species present during sequencing. The expression abundance levels of 10 species reached at least 2% in one sample, while other species were classified into unclassified categories (Table S2). Similarly, OTUs were calculated for the fungal species with three replications for every sample; the list of these species is provided in Table S3.

### Bacterial Communities in Rhizosphere Soil After *T. foetida* Inoculation

**Fig. 1:**
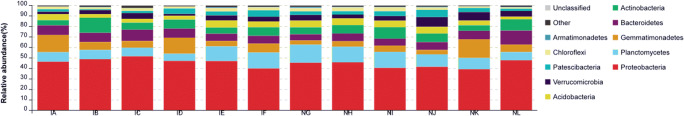
Bacterial compositions of the different communities in rhizosphere soil. Relative abundance levels of different bacterial phyla within different libraries. IA (*T. foetida* + 1.5% RD of Jianzhuang), IB (*T. foetida* + 3% RD of Jianzhuang), IC (*T. foetida* + 5% RD of Jianzhuang), ID (*T. foetida* + RD of Jianzhuang), IE (*T. foetida* + 1.5 times RD of Jianzhuang), IF (*T. foetida* + no Jianzhuang), NG (mock + 1.5% RD of Jianzhuang), NH (mock + 3% RD of Jianzhuang), NI (mock + 5% RD of Jianzhuang), NJ (mock + % RD of Jianzhuang), NK (mock + 1.5 times RD of Jianzhuang), and NL (mock + no Jianzhuang). RD stands for recommended dose, and mock means ddH_2_O application

There were differences in the OTUs within *T. foetida-*infected and mock libraries of Jianzhuang-coated seeds. The relative abundance of different microbial communities varied in the *T. foetida-*infected and mock samples. The results showed that the IC (5% RD) library had maximum abundance, while the IF (no seed treatment) and NK (mock of 1.5 times RD) libraries had minimum relative abundance of *Proteobacteria*. However, the overall abundance of *Proteobacteria* was high in *T. foetida-*infected libraries. Similarly, the relative abundance of *Planctomycetes* was high in the mock group, including the NG (1.5% RD), NH (3% RD), NI (5% RD), and NJ (RD) groups, compared to the *T. foetida*-infected group, including the IA (1.5% RD), IB (3% RD), IC (5% RD), and ID (RD) samples. Additionally, the abundance levels of all other bacterial phyla were different under different treatments (Fig. 1).

### Fungal Communities in Rhizosphere Soil After *T. foetida* Infection

**Fig. 2:**
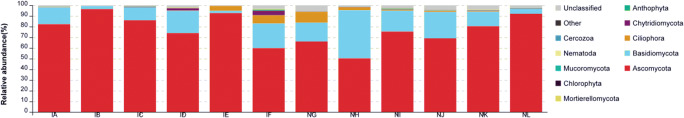
Fungal compositions of the different communities in rhizosphere soil. Relative abundance levels of different fungal phyla within different samples. IA (*T. foetida* + 1.5% RD of Jianzhuang), IB (*T. foetida* + 3% RD of Jianzhuang), IC (*T. foetida* + 5% RD of Jianzhuang), ID (*T. foetida* + RD of Jianzhuang), IE (*T. foetida* + 1.5 times RD of Jianzhuang), IF (*T. foetida* + no Jianzhuang), NG (mock + 1.5% RD of Jianzhuang), NH (mock + 3% RD of Jianzhuang), NI (mock + 5% RD of Jianzhuang), NJ (mock + % RD of Jianzhuang), NK (mock + 1.5 times RD of Jianzhuang), and NL (mock + no Jianzhuang). RD stands for recommended dose, and mock means ddH_2_O application

Fungal communities were analyzed after *T. foetida* infection in Jianzhuang-coated seeds using OTU analysis (Table S3). The *T. foetida*-infected samples from the IB (3% RD) library had the maximum number of *Ascomycota*. The relative abundance of *Basidiomycota* was higher in the mock-infected libraries than in the *T. foetida-*infected libraries. The maximum abundance of *Basidiomycota* was found in the NH (mock of 3% RD) library, and the minimum abundance of *Basidiomycota* was found in the infected IE (1.5% RD) library, suggesting that Jianzhuang (1.5% RD) was better for controlling *T. foetida* infection. Additionally, the abundance levels of all other fungal phyla were different under different treatments (Fig. 2).

### Cluster and Principal Component Analysis for Bacterial Communities

**Fig. 3:**
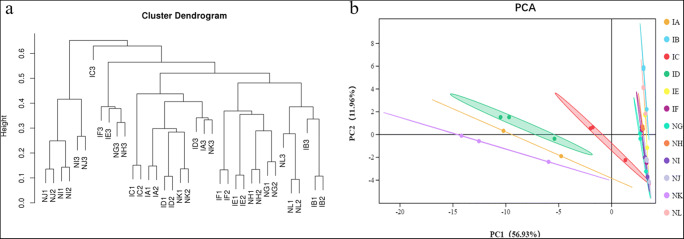
Beta (β) diversity of twelve bacterial samples. **a** Phylogenetic tree. **b** Two-dimensional PCoA analysis. IA (*T. foetida* + 1.5% RD of Jianzhuang), IB (*T. foetida* + 3% RD of Jianzhuang), IC (*T. foetida* + 5% RD of Jianzhuang), ID (*T. foetida* + RD of Jianzhuang), IE (*T. foetida* + 1.5 times RD of Jianzhuang), IF (*T. foetida* + no Jianzhuang), NG (mock + 1.5% RD of Jianzhuang), NH (mock + 3% RD of Jianzhuang), NI (mock + 5% RD of Jianzhuang), NJ (mock + % RD of Jianzhuang), NK (mock + 1.5 times RD of Jianzhuang), and NL (mock + no Jianzhuang). RD stands for recommended dose, and mock means ddH_2_O application

As shown in Fig. 3a, comparing the distances between the six treatments and their control, it can be seen for the two groups, 5% “Jianzhuang” dressing (IC) and its control (NI) and 1 times “Jianzhuang” dressing (ID) and its control (NJ), the treatment group is the farthest from the control group. This was followed by 1.5% “Jianzhuang” seed dressing (IA) and its control (NG) and 1.5 times “Jianzhuang” seed dressing (IE) and its control (NK). Among the two groups of 3% “Jianzhuang” seed dressing (IB) treatment and its control (NH), and no seed treatment (IF) and its control (NL), the distance between the treatment and the control is relatively small.

Based on the unweighted UniFrac distance metrics, PCoA was performed to assess the similarities of different infected and mock libraries in Jianzhuang-coated seeds. The PCoA analysis results revealed maximum variations of 56.93% (PC1) and 11.96% (PC2), as shown in Fig. 3b. Similar results were also obtained between the 3% “Jianzhuang” seed dressing (IB) treatment and the control (NH), the no seed treatment group (IF) and the control (NL). The distance between these two groups was small, and the distance between the other groups was large. We also found that among all of the inoculated treatment groups, IA (1.5% RD), IC (5% RD), and ID (RD) were farther away from the other treatments, while the distances between IB (3% RD) and IE (1.5 times RD) were closer, indicating that the effects of these two concentrations of Jianzhuang seed dressings on the rhizosphere bacterial community were similar.

### Cluster and Principal Component Analysis of Fungal Communities

**Fig. 4:**
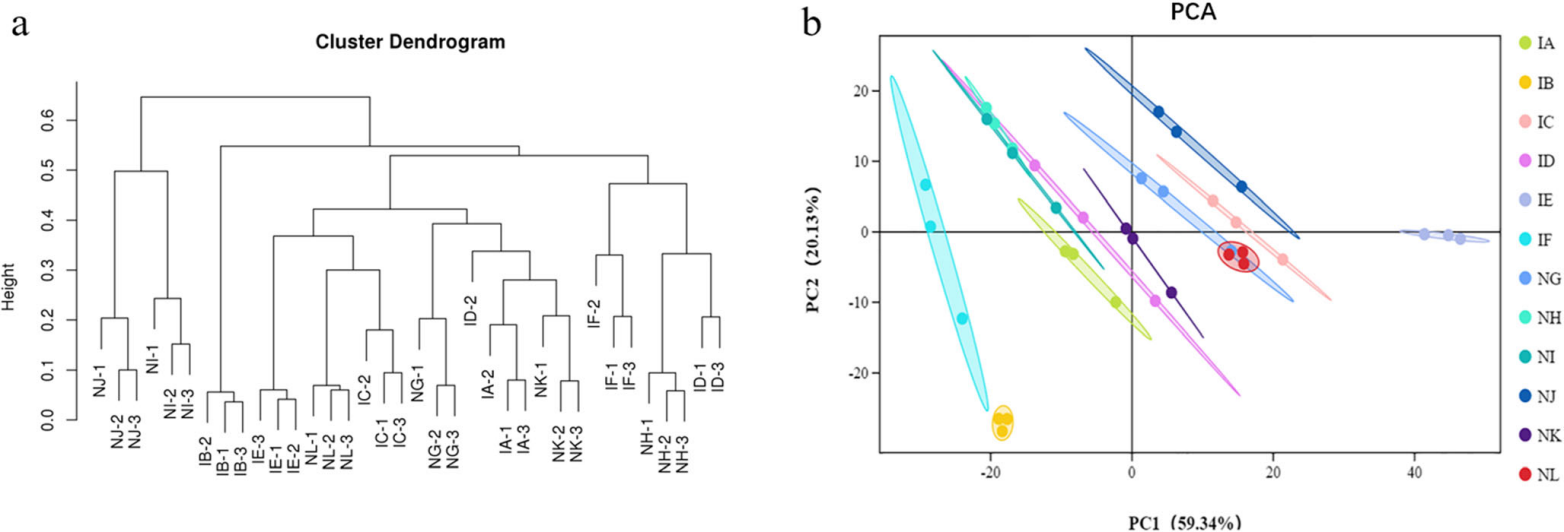
Beta (β) diversity of twelve fungal samples. **a** Phylogenetic tree. **b** Two-dimensional PCoA analysis. IA (*T. foetida* + 1.5% RD of Jianzhuang), IB (*T. foetida* + 3% RD of Jianzhuang), IC (*T. foetida* + 5% RD of Jianzhuang), ID (*T. foetida* + RD of Jianzhuang), IE (*T. foetida* + 1.5 times RD of Jianzhuang), IF (*T. foetida* + no Jianzhuang), NG (mock + 1.5% RD of Jianzhuang), NH (mock + 3% RD of Jianzhuang), NI (mock + 5% RD of Jianzhuang), NJ (mock + % RD of Jianzhuang), NK (mock + 1.5 times RD of Jianzhuang), and NL (mock + no Jianzhuang). RD stands for recommended dose, and mock means ddH_2_O application

In Fig. 4a, comparing the distances between the 6 treatments and their mock, the distance between the 3% “Jianzhuang” seed dressing (IB) treatment and the mock (NH) treatment was the largest. Among the three groups of 5% “Jianzhuang” seed dressing (IC) and its mock (NI), 1 time of “Jianzhuang” seed dressing (ID) and its mock (NJ), and no seed treatment (IF) and its mock (NL), the distance between the treatments and their mock was slightly shorter. Among 1.5% “Jianzhuang” seed dressing (IA) and its mock (NG) and 1.5 times “Jianzhuang” seed dressing (IE) and its mock (NK), the difference between the treatment group and the control group was relatively small.

The results of PCoA revealed maximum variations of 59.34% (PC1) and 20.13% (PC2). It can be seen from Fig. 4b that both the treatment group and the mock group have good repeatability, and the sample is in a confidence circle. At the same time, the distance between each group and their mock was large, indicating that *T. foetida* has a relatively large impact on the fungal community of wheat rhizosphere soil.

### Heatmap Analysis for Bacterial Communities

**Fig. 5:**
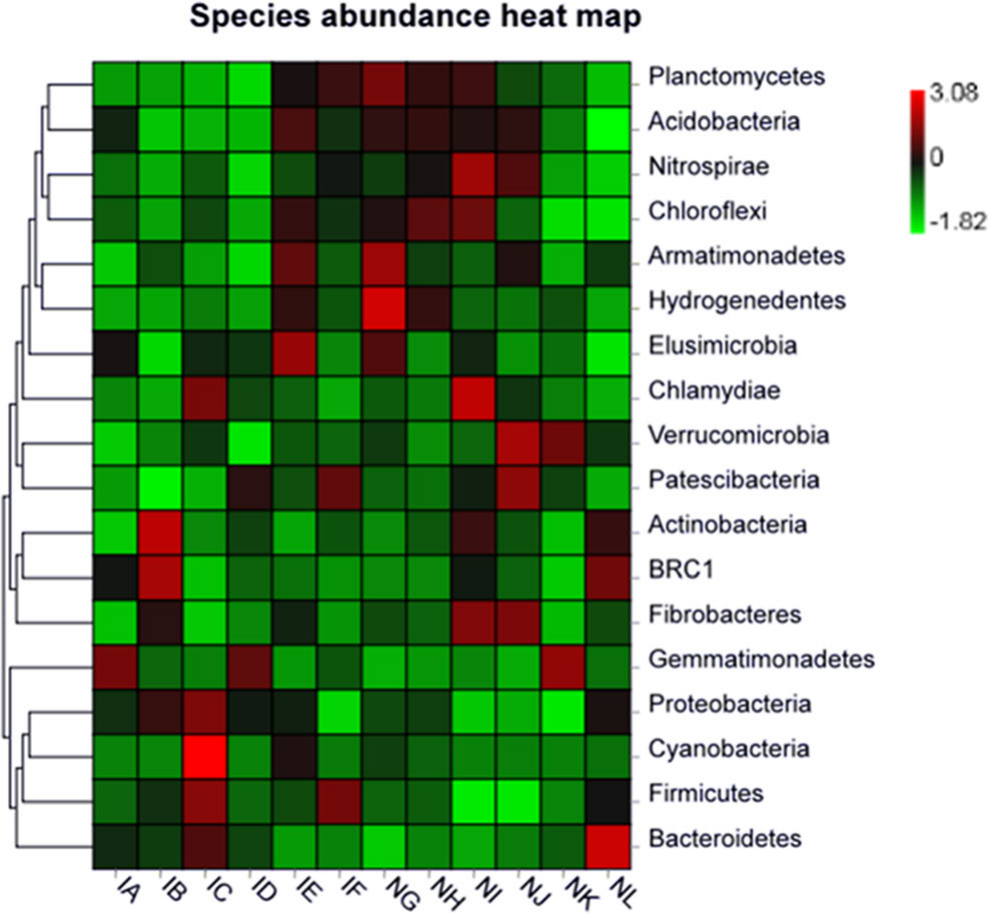
Profiling results for the twelve samples. Richness heatmap of the 18 most abundant bacterial phyla. IA (*T. foetida* + 1.5% RD of Jianzhuang), IB (*T. foetida* + 3% RD of Jianzhuang), IC (*T. foetida* + 5% RD of Jianzhuang), ID (*T. foetida* + RD of Jianzhuang), IE (*T. foetida* + 1.5 times RD of Jianzhuang), IF (*T. foetida* + no Jianzhuang), NG (mock + 1.5% RD of Jianzhuang), NH (mock + 3% RD of Jianzhuang), NI (mock + 5% RD of Jianzhuang), NJ (mock + RD of Jianzhuang), NK (mock + 1.5 times RD of Jianzhuang), and NL (mock + no Jianzhuang). RD stands for recommended dose, and mock means ddH_2_O application

The abundance levels of eighteen bacterial phyla were characterized in *T. foetida*-inoculated and mock libraries. Community heatmap analysis was scaled to demonstrate the abundance levels of the various bacterial communities. The results showed that *Proteobacteria* (IC, *T. foetida* infected + 5% RD), *Actinobacteria* (IB, *T. foetida* infected + 3% RD), *Firmicutes* (IC, *T. foetida* infected + 5% RD), BRC1 (IB, *T. foetida* infected + 3% RD), *Cyanobacteria* (IC, *T. foetida* infected + 5% RD), and *Elusimicrobia* (IE, *T. foetida* infected + 1.5 times of RD) were abundant in the *T. foetida* infected compared to mock samples, while *Planctomycetes* (NG, mock + 1.5% RD), *Bacteroidetes* (NL, mock + no Jianzhuang treatment), *Verrucomicrobia* (NJ, mock + RD), *Patescibacteria* (NJ, mock + RD), *Chloroflexi* (NI, mock + 5% RD), *Armatimonadetes* (NG, mock + 1.5% RD), *Nitrospirae* (NI, mock + 1.5% RD), *Fibrobacteres* (NI, mock + 1.5% RD), *Chlamydiae* (NI, mock + 1.5% RD), and *Hydrogenedentes* (NG, mock + 1.5% RD) were abundant in the mock compared to *T. foetida*-infected libraries. However, *Gemmatimonadetes* (NK (1.5 times of RD), ID (RD), and IA (1.5% RD)) and *Acidobacteria* had similar abundances in *T. foetida*-infected and mock samples (Fig. 5).

### Heatmap Analysis for Fungal Communities

**Fig. 6:**
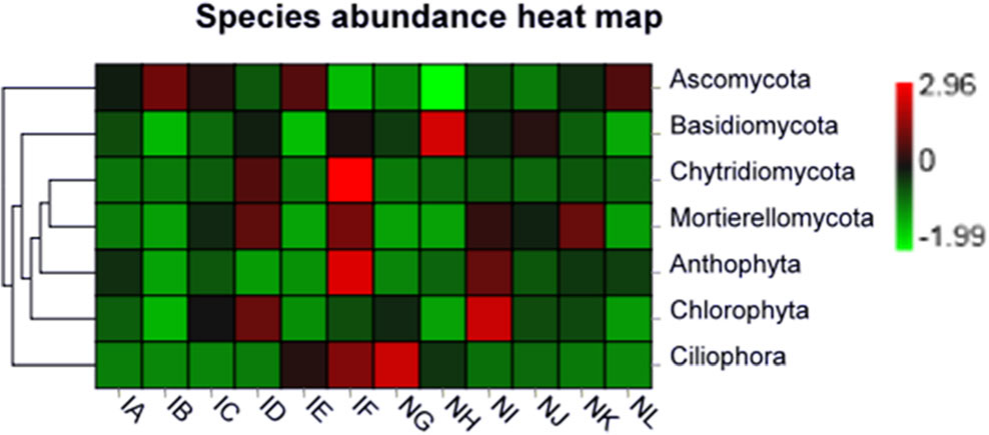
Profiling results of the twelve samples. Richness heatmap of the 7 most abundant fungal phyla. IA (*T. foetida* + 1.5% RD of Jianzhuang), IB (*T. foetida* + 3% RD of Jianzhuang), IC (*T. foetida* + 5% RD of Jianzhuang), ID (*T. foetida* + RD of Jianzhuang), IE (*T. foetida* + 1.5 times RD of Jianzhuang), IF (*T. foetida* + no Jianzhuang), NG (mock + 1.5% RD of Jianzhuang), NH (mock + 3% RD of Jianzhuang), NI (mock + 5% RD of Jianzhuang), NJ (mock + % RD of Jianzhuang), NK (mock + 1.5 times RD of Jianzhuang), and NL (mock + no Jianzhuang). RD stands for recommended dose, and mock means ddH_2_O application

The abundance levels of seven fungal phyla were characterized into *T. foetida-*infected and mock libraries. The results revealed that only *Ascomycota* (IB = 3% RD) was abundant in the *T. foetida*-infected libraries, while *Basidiomycota* (NH = 3 RD), *Ciliophora* (NG, mock + 1.5% RD), *Chytridiomycota* (IF, *T. foetida* infected with no Jianzhuang treatment), *Anthophyta* (IF, *T. foetida* infected with no Jianzhuang treatment), and *Chlorophyta* (NI, mock + 5% RD) were more abundant in mock libraries than in *T. foetida*-infected libraries. However, *Mortierellomycota* had similar abundance in *T. foetida*-infected (ID, *T. foetida* + RD) and mock (NK, mock + 1.5 times RD) libraries (Fig. 6).

### Alpha Diversity Analysis of Bacterial and Fungal Communities in Wheat Rhizosphere Soil

**Fig. 7:**
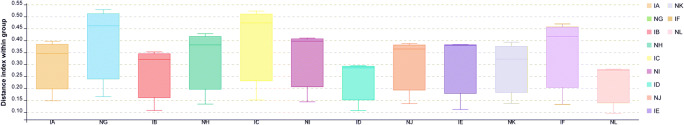
Alpha (α) diversity levels of twelve bacterial samples. IA (*T. foetida* + 1.5% RD of Jianzhuang), IB (*T. foetida* + 3% RD of Jianzhuang), IC (*T. foetida* + 5% RD of Jianzhuang), ID (*T. foetida* + RD of Jianzhuang), IE (*T. foetida* + 1.5 times RD of Jianzhuang), IF (*T. foetida* + no Jianzhuang), NG (mock + 1.5% RD of Jianzhuang), NH (mock + 3% RD of Jianzhuang), NI (mock + 5% RD of Jianzhuang), NJ (mock + % RD of Jianzhuang), NK (mock + 1.5 times RD of Jianzhuang), and NL (mock + no Jianzhuang). RD stands for recommended dose, and mock means no *T. foetida* application

PCoA analysis provided little variability information, and distance index within-group analysis was further used to assess the distance within groups. The results showed that the IB (3% RD), NI (mock of 5% RD), ID (RD), NJ (mock of RD), and NL (mock of no Jianzhuang treatment) libraries had lower distances within the groups (Fig. 7).

**Fig. 8:**
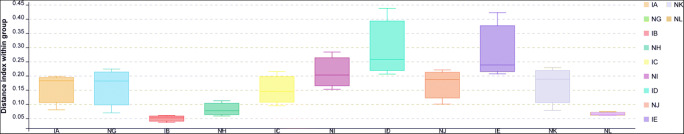
Alpha (α) diversity levels of twelve fungal samples. IA (*T. foetida* + 1.5% RD of Jianzhuang), IB (*T. foetida* + 3% RD of Jianzhuang), IC (*T. foetida* + 5% RD of Jianzhuang), ID (*T. foetida* + RD of Jianzhuang), IE (*T. foetida* + 1.5 times RD of Jianzhuang), IF (*T. foetida* + no Jianzhuang), NG (+ 1.5% RD of Jianzhuang), NH (mock + 3% RD of Jianzhuang), NI (mock + 5% RD of Jianzhuang), NJ (mock + % RD of Jianzhuang), NK (mock + 1.5 times RD of Jianzhuang), and NL (mock + no Jianzhuang). RD stands for recommended dose, and mock means application of ddH_2_O instead of *T. foetida*

Alpha (α) diversity analysis was also performed for fungal communities. The results showed that the IB (3% RD), NH (mock of 3% RD), IE (1.5 times RD), and NL (mock of no Jianzhuang treatment) libraries had minimum distances within the groups compared to the other samples (Fig. 8).

## Discussion

In this study, we investigated the effects of *T. foetida* on the microbial community with different concentrations of Jianzhuang pesticide-coated seeds and identified some communities that may contribute to the control of wheat common bunt, which will provide some important information on the interaction of the rhizosphere community with plant growth.

The raw reads, clean reads, raw tags, clean tags, effective tags, and effective ratio (%) were calculated for each treatment (Table S1) and were consistent with previous reports [33]. *T. foetida*-infected samples with Jianzhuang at different concentrations had slightly higher *Proteobacteria* and *Gemmatimonadetes* relative abundance levels than the mock libraries (Fig. 1). These findings suggest that Jianzhuang increased *Proteobacteria* diversity, which was affected by *T. foetida*. Previous studies revealed that *Proteobacteria* has roles in plant disease suppression and gene regulation, which have plant growth-promoting activities [34–36]. Interestingly, *T. foetida* belongs to the phylum *Basidiomycota*, and *T. foetida*-infected samples had a slightly lower abundance of *Basidiomycota*, possibly because of Jianzhuang application [37]. However, the relative abundance of the phylum *Ascomycota* was greater in the *T. foetida*-infected samples (Fig. 2) [38]. The observed results in this experiment revealed that *T. foetida* might increase the abundance levels of some fungal communities.

Based on the unweighted UniFrac distance metric, cluster and PCoA analyses were conducted to evaluate the similarities among the *T. foetida*-infected and mock libraries [39]. The results of cluster analysis (Fig. 3a) and PCoA analysis (Fig. 3b) showed that replicate samples of the same treatment under the same concentration of Jianzhuang were similar. Additionally, similar results were observed for the fungal phyla during cluster and PCoA analysis (Fig. 4a, b). The richness levels of eighteen bacterial phyla were checked in the *T. foetida*-infected and mock libraries. *Actinobacteria* and *Cyanobacteria* are able to suppress different plant pathogens and promote the morpho-physiological attributes of plants [40, 41]. Our results revealed that the richness levels of *Actinobacteria* and *Cyanobacteria* were high in *T. foetida*-infected plants, suggesting that Jianzhuang activates *Actinobacteria* and *Cyanobacteria* richness. Additionally, *Nitrospirae* abundance was high in mock samples (Fig. 5). Previous results reported that Oehmen played an important role in nitrogen fixation and removal [42]. In our results, *Nitrospirae* abundance was low, suggesting that *T. foetida* might have a negative impact on it. Similarly, the richness levels of seven fungal phyla were demonstrated in the *T. foetida*-infected and mock samples. The richness of *Basidiomycota* was abundant in the NH (3% RD) sample. *T. foetida* belongs to the phylum *Basidiomycota*, which suggests that Jianzhuang inhibits the proliferation of *T. foetida* (Fig. 6).

Advances in the knowledge of rhizosphere soil microbial diversity depend on improved approaches [43]. Soil microorganisms play important roles in soil fertility and soil biochemical processes and regulate soil temperature [44–48]. It is acknowledged that traditional molecular biological techniques such as simple PCR, terminal restriction fragment-length polymorphism (T-RFLP), and denaturing gradient gel electrophoresis (DGGE) have their own forms of reference and have intense labor requirements [49]. Next-generation sequencing has been described as easier and more accurate than traditional molecular techniques in characterizing rhizosphere soil microorganisms under controlled and natural conditions. Plant morpho-physiological attributes and performance are highly associated with plant-associated soil microorganisms under different conditions [50]. Up to a few thousand different species of bacteria, fungi, and various protists, including plant roots, act as biocontrol agents against various plant pathogens [50–53].

Previous studies have found that microbial abundance was greater in infected samples than in mock samples [7, 54]. The results of this study suggested that inoculation with *T. foetida* had negative effects on the rhizosphere soil microbial community by increasing or decreasing their relative abundance levels. The abundance levels of the bacterial communities *Verrucomicrobia*, *Patescibacteria*, *Armatimonadetes*, *Nitrospirae*, *Fibrobacteres*, *Chlamydiae*, and *Hydrogenedentes* and the fungal communities *Basidiomycota* and *Ciliophora* were higher in the mock group than in the *T. foetida*-infected group, which may contribute to the control of wheat common bunt.

## Supplementary Information

Figure S1 Molecular detection of *T. foetida* from leaf samples with specific primers. Lines 1 and 25 negative controls; lines 2 and 26 positive controls; lines 3-24 and lines 27-40 *T. foetida*-infected leaf samples; M, DL2000 Marker (100, 250, 500, 750, 1000, 2000 bp); black arrows show the target band of 660 bp. (PNG 481 kb)

High Resolution Image (TIF 5294 kb)

Table S1 Raw reads, clean reads, raw tags, clean tags, effective tags, and effective ratio (%) of twelve samples. (XLSX 13 kb)

Table S2 OTUs of bacterial communities of twelve samples with three replications (XLSX 4825 kb)

Table S3 OTUs of fungal communities of twelve samples with three replications (XLSX 691 kb)
