# Supplementary figures and images for: Effects of Tilletia foetida on Microbial Communities in the Rhizosphere Soil of Wheat Seeds Coated with Different Concentrations of Jianzhuang
Source: Microb Ecol. 2021 Feb 1;82(3):736–45. doi: 10.1007/s00248-021-01696-w (PMC8463399; doi:10.1007/s00248-021-01696-w)

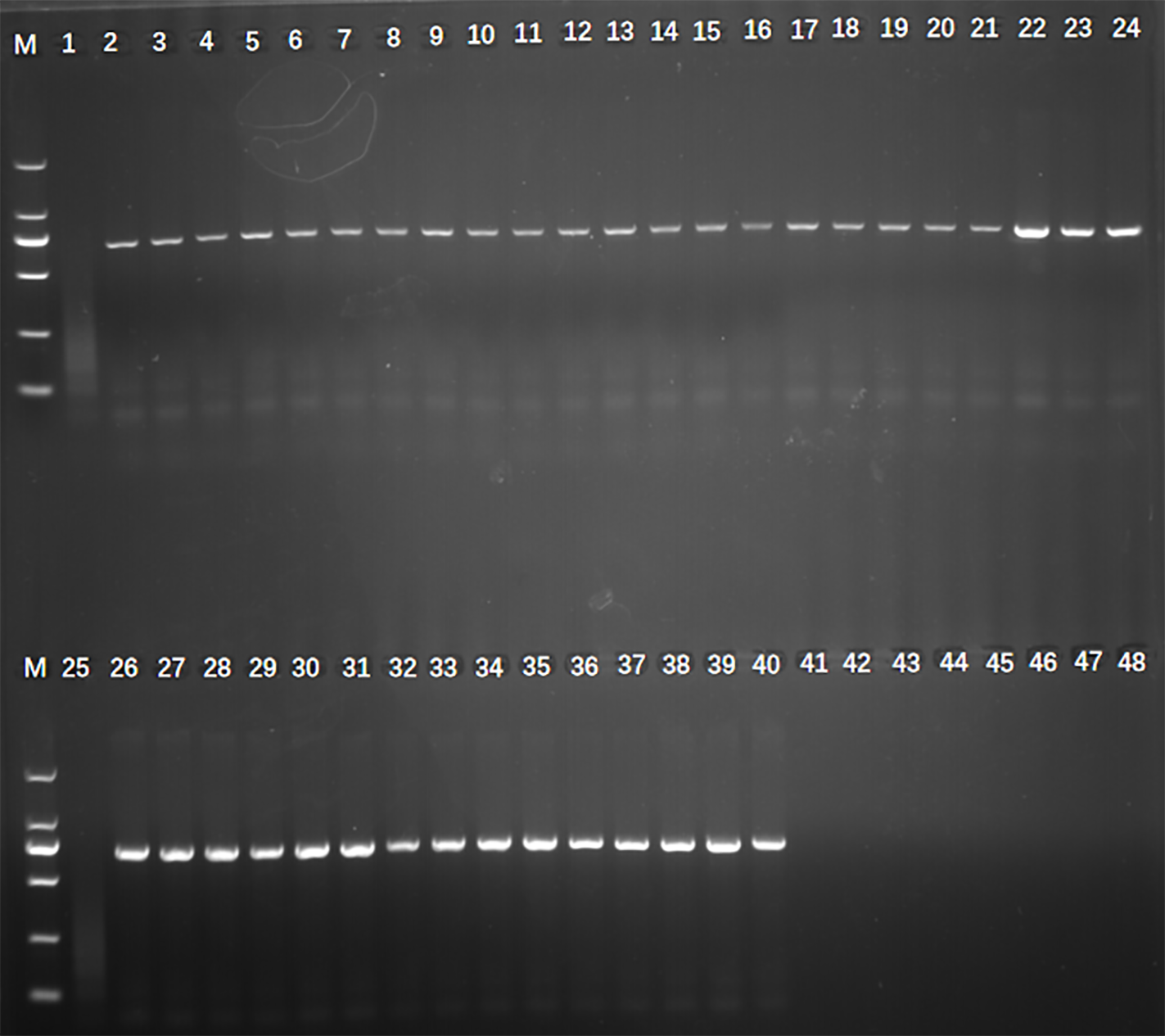

Supplement: Supplementary file 1 — Molecular detection of T. foetida from leaf samples with specific primers. Lines 1 and 25 negative controls; lines 2 and 26 positive controls; lines 3-24 and lines 27-40 T. foetida-infected leaf samples; M, DL2000 Marker (100, 250, 500, 750, 1000, 2000 bp); black arrows show the target band of 660 bp. (PNG 481 kb) [file 248_2021_1696_Fig9_ESM.png]

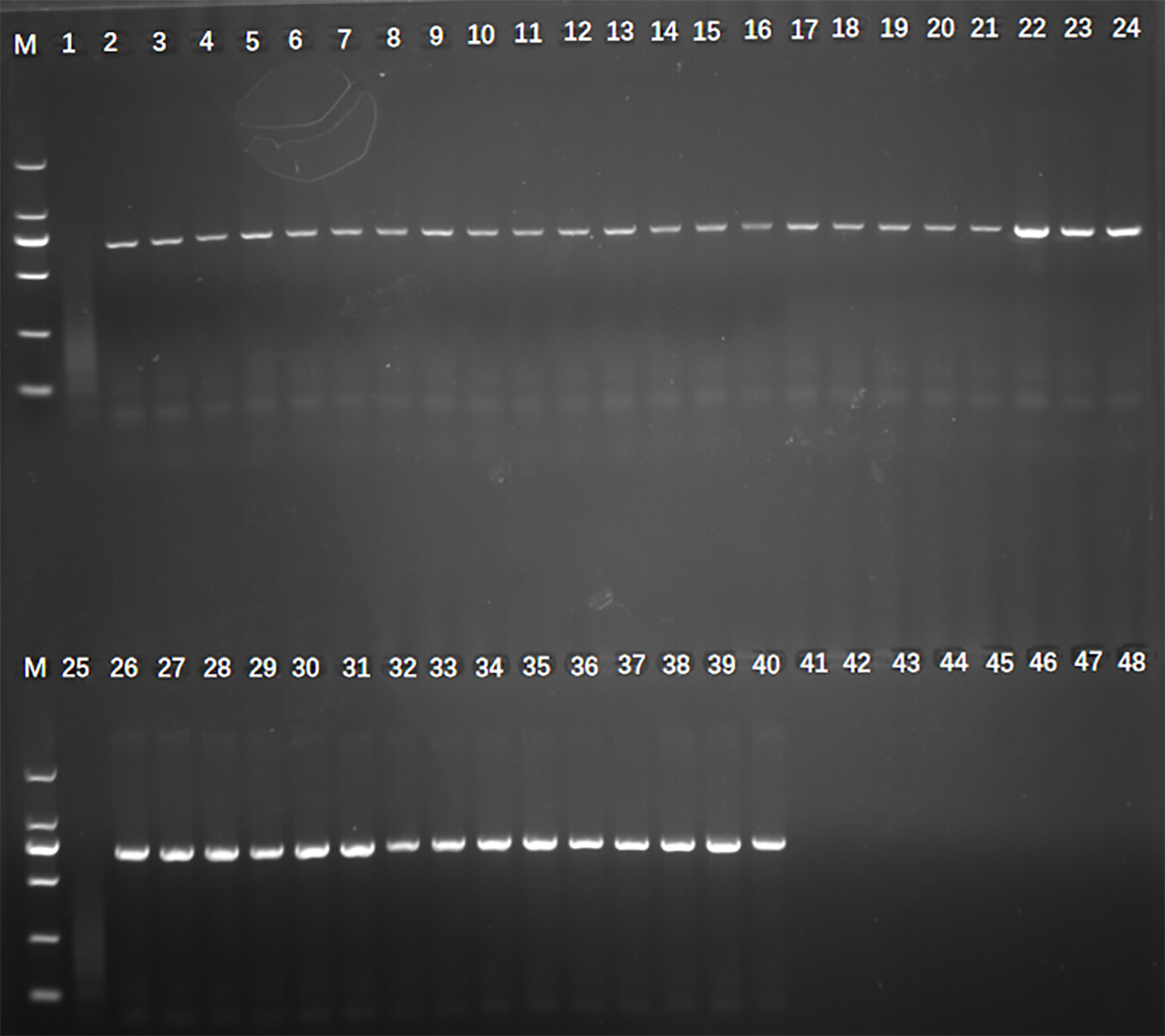

Supplement: Supplementary file 2 — High Resolution Image (TIF 5294 kb) [file 248_2021_1696_MOESM1_ESM.tif]
